# Risk factors associated with Tuberculosis (TB) among people living with HIV/AIDS: A pair-matched case-control study in Guangxi, China

**DOI:** 10.1371/journal.pone.0173976

**Published:** 2017-03-30

**Authors:** Zhezhe Cui, Mei Lin, Shaofa Nie, Rushu Lan

**Affiliations:** 1 Guangxi Zhuang Autonomous region center for disease prevention and control, Guangxi, China; 2 Department of Epidemiology and Statistics, Public Health School, Tongji Medical College, Huazhong University of Science and Technology, Wuhan, China; Institut Pasteur of Shanghai Chinese Academy of Sciences, CHINA

## Abstract

**Background:**

As one of the poorest provinces in China, Guangxi has a high HIV and TB prevalence, with the annual number of TB/HIV cases reported by health department among the highest in the country. However, studies on the burden of TB-HIV co-infection and risk factors for active TB among HIV-infected persons in Guangxi have rarely been reported.

**Objective:**

To investigate the risk factors for active TB among people living with HIV/AIDS in Guangxi Zhuang autonomous region, China.

**Methods:**

A surveillance survey was conducted of 1 019 HIV-infected patients receiving care at three AIDS prevention and control departments between 2013 and 2015. We investigated the cumulative prevalence of TB during 2 years. To analyze risk factors associated with active TB, we conducted a 1:1 pair-matched case-control study of newly reported active TB/HIV co-infected patients. Controls were patients with HIV without active TB, latent TB infection or other lung disease, who were matched with the case group based on sex and age (± 3 years).

**Results:**

A total of 1 019 subjects were evaluated. 160 subjects (15.70%) were diagnosed with active TB, including 85 clinically diagnosed cases and 75 confirmed cases. We performed a 1:1 matched case-control study, with 82 TB/HIV patients and 82 people living with HIV/AIDS based on surveillance site, sex and age (±3) years. According to multivariate analysis, smoking (*OR* = 2.996, 0.992–9.053), lower CD 4^+^ T-cell count (*OR* = 3.288, 1.161–9.311), long duration of HIV-infection (*OR* = 5.946, 2.221–15.915) and non-use of ART (*OR* = 7.775, 2.618–23.094) were independent risk factors for TB in people living with HIV/AIDS.

**Conclusion:**

The prevalence of active TB among people living with HIV/AIDS in Guangxi was 173 times higher than general population in Guangxi. It is necessary for government to integrate control planning and resources for the two diseases. Medical and public health workers should strengthen health education for TB/HIV prevention and treatment and promote smoking cessation. Active TB case finding and early initiation of ART is necessary to minimize the burden of disease among patients with HIV, as is IPT and infection control in healthcare facilities.

## Introduction

Tuberculosis (TB) and human immunodeficiency virus (HIV) co-infection is a severe public health problem around the world [1–2]. Beginning in the 1980’s, the epidemic of AIDS has accelerated the rates of transmission and mortality due to TB. The rate of TB/HIV co-infection peaked in the 1990’s. With the implementation of antiretroviral treatment (ART), the incidence of TB has decreased slowly. Unfortunately, globalization with economic and cultural exchanges has contributed disease spread in recent years[3]. The increased trend of TB/HIV co-infection is approximately 10% per year[4]. In 2014, almost 1.2 million cases (12.5%) of TB worldwide were associated with HIV infection, and TB accounts for an estimated 350,000 deaths among HIV-infected persons. Asia has a high-prevalence area of TB/HIV co-infection second to Africa[5].

The prevalence of HIV infection in the Asia-Pacific region (including China) is at medium level (0.09%). The number of HIV/AIDS cases in China has increased since the first case was identified in 1985, although the prevalence rate is relatively low (< 0.09%). However, the burden of TB in China is heavy. According to World Health organization (WHO)[4], the TB cases reported from China was the third highest globally in 2014, following India and Indonesia. As one of the poorest provinces in China, Guangxi is also regarded as a high HIV and TB prevalence area, where the annual number of TB/HIV cases reported by health department is the largest in China[6–7]. However, studies of the burden of TB-HIV co-infection and the risk factors for active TB among HIV-infected persons in Guangxi have rarely been reported. For this reason, we investigated the prevalence and risk factors for active TB among people living with HIV/AIDS receiving care in three AIDS prevention and control departments in higher HIV prevalence cities in Guangxi.

## Methods

### Study population and diagnostic methods

**Fig 1:**
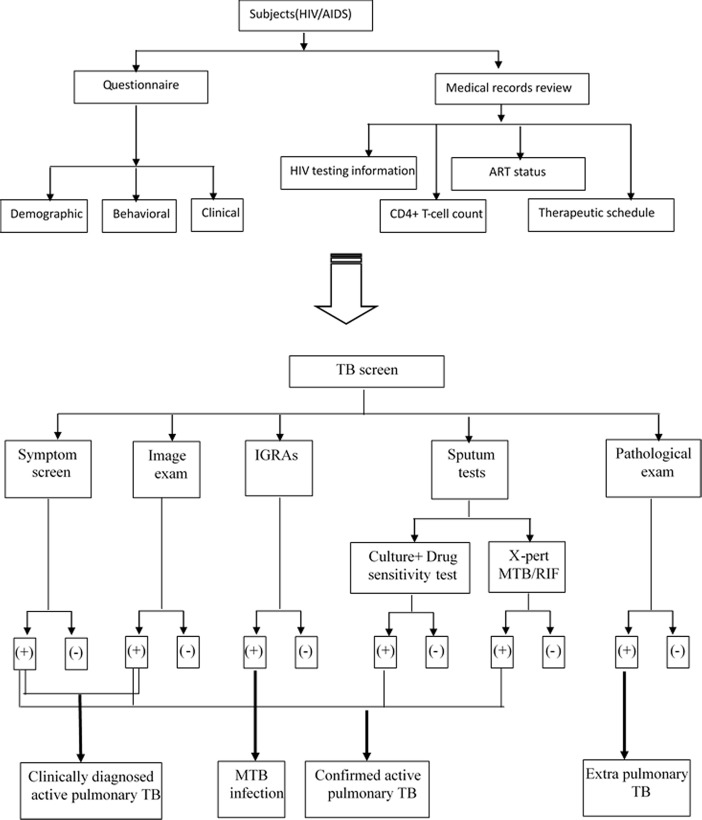
The program of information collection and TB screen among HIV/AIDS.

The study population was composed of 1 019 people living with HIV/AIDS who received care at three AIDS prevention and control departments between 2013 and 2015. A surveillance survey was conducted using questionnaire(collecting demographic behavioral and clinical characteristics), medical records review(collecting HIV testing information, latest CD4+ T-cell count, ART status and therapeutic schedule) and TB screening[including symptom screen, image examination (IE), Interferon-Gamma Release Assays (IGRAs)[8], sputum culture, drug sensitivity testing and Xpert MTB/RIF Assay[9]. Unfortunately, we haven’t access to the information of viral load determination because of the shortage of reagent at the time of the study. MTB infection case was defined by IGRAs positive. Clinically diagnosed active TB case was defined by IE and TB symptom positive. Confirmed active TB was diagnosed by direct detection methods, including sputum culture or Xpert MTB/RIF Assay or histopathological examination of non-sputum specimens. All study patients provided three sputum samples (one spot sample, one morning sample and another evening sample) for bacteriological examination and whole blood (more than 4 ml) for IGRAs. (Fig 1).

### Study design and variables

We analyzed the cumulative prevalence of active TB (included clinically diagnosed and confirmed cases) among 1 019 people living with HIV/AIDS during the study period. To survey factors associated with occurrence of active TB in people living with HIV/AIDS, we performed a 1:1 matched case-control study. The case group included 82 HIV-infected patients with active TB, after exclusion of 10 subjects who were unwilling, 8 subjects unable to participate in this project with mental disease or severe complication, 1 subjects who died at the time being diagnosed active TB and 59 subjects who could not be matched 1:1 with controls in their surveillance site. All subjects in the case group had TB newly diagnosed during the research period. The control group consisted of 82 HIV-infected patients without active TB or latent mycobacterium tuberculosis infection (LTBI) or other lung disease, who were matched with the case group based on surveillance site, sex and age (±3) years for avoiding confounding.

The following variables were also assessed: demographic characteristics (occupation, Ethnicity, education status, marital status, place of residence, local residence duration, household income per capita, number of family members), behavioral characteristics (history of close contact with TB patient, history of BCG vaccination, history of former plasma donors, history of intravenous drug abuse, HIV infection status of regular sex partner, extramarital sex, condom use, history of alcohol use, history of cigarette smoking), clinical characteristics [body mass index (BMI), chronic disease, latest CD 4^+^ T-cell count, HIV risk factors, use of ART, duration of HIV infection].

### Statistical analysis

Categorical data were analyzed using independent sample *Chi-square* tests and Continuous variables were analyzed using independent sample *t* tests. A conditional logistic regression analysis was used to avoid effects of confounding variables for active TB. For this analysis, those variables with *P* < 0.05 on the univariate analysis were entered into the multivariate analysis model. For stepwise multivariate analysis, if there was significant correlation between independent variables, only one was entered into the model to avoid multicollinearity. Variables with a significant association in bivariate analysis were included in a final logistic regression model. All *P* values were two-tailed, and *P* < 0.05 was considered statistically significant. Odds ratios (*OR*) and their 95% confidence intervals (CIs) were estimated using conditional logistic regression, with TB as an outcome. All of the statistical analyses were performed using Epi-Info (version 6, CDC, Atlanta, GA) and IBM SPSS statistical data editor (version 19, Statistical Product and Service Solutions, Chicago, IL).

This research was approved by the Institutional Review Board of the Guangxi (IRB 0001594, FWA 00001359). Ethics committees approve this consent procedure. All participants provided their written informed consent to participate in this study.

## Results

### Population of TB Surveillance

The mean age of the 1 019 HIV-infected patients enrolled was 48.97±14.44 years (mean ± standard deviation) (range 19~85). Sex ratio was 2.33: 1 (male/female). The majority of subjects reported their occupation as farmers (61.3%), followed by unemployed (14.3%). The median of household income per capita was 5 582.63 Yuan per year (range 77~50 000 Yuan py). The median of BMI was 20.46 (range 12.3–41.7). The median of latest CD 4^+^ T-cell count was 235.68 cells/mm^3^ (range 2–1610). The percentage of individuals who reported opportunistic infection symptoms was 26.4%. Only 47.5% of the subjects had received ART before the study period (Table 1).

**Table 1:** HIV/AIDS cohort description.

Characteristic	Subjects(n = 1 019) mean±SD or n(%)
Gender	
Male	713(69.97)
Female	306(30.03)
Age(years)	48.97±14.44
Ethnicity	
Han	590(57.90)
Zhuang	418(41.02)
Other	11(1.08)
Education status	
Illiterate	102(10.01)
Primary school	409(40.14)
Junior middle school	414(40.62)
Above high school	94(9.22)
Marital status	
Married	735(72.13)
other	284(27.87)
Household incomes per capita,Yuan	5 582.63±4881.30
BMI	20.46±2.87
Latest CD 4^+^ T-cell count, cells/mm^3^	235.68±153.26
Status of ART	
Have access to ART	484(47.50)
Haven't access to ART	535(52.50)
Opportunistic infections symptoms	
Yes	269(26.40)
No	750(73.60)

### Cumulative prevalence of active tuberculosis among people living with HIV/AIDS

Over a total of 1 019 of subjects, 160 subjects (15.70%) were diagnosed with active TB, including 85 clinically diagnosed cases (1 patient at the time being diagnosed active TB) and 75 confirmed cases (72 pulmonary TB and 3 extrapulmonary). The detection rate of latent mycobacterium tuberculosis infection (LTBI) was 15.30% detected by IGRAs. In addition, we found 54 subjects with NTM infection.

### Characteristics and risk factors for active tuberculosis among people living with HIV/AIDS

In this case-control study, the mean age of HIV-infected individuals with active TB was 47.95 ± 14.13 years, and 89.02% of the subjects were male. The mean duration of HIV-infection was 7.30 ± 3.53 years. The mean of BMI was 19.19 ± 2.46. The mean of latest CD 4^+^ T-cell count was 188.78 ± 235.95 cells/mm^3^ (Table 2).

**Table 2:** Baseline characteristic of cases and controls.

Characteristic	Cases(n = 82) mean±SD or n(%)	Controls(n = 82) mean±SD or n(%)
Age at baseline, years	47.95 ± 14.13	48.04±14.18
Sex		
Male	73(89.02)	73(89.02)
Female	9(10.98)	9(10.98)
Duration of HIV-infection, years	7.30±3.53	6.09±3.09
BMI, kg/m^3^	19.19±2.46	19.70±2.69
Latest CD 4^+^ T-cell count, cells/mm^3^	188.78±235.95	217.66±189.93

On univariate analysis of demographic variables, there were no statistically significant variables associated with active TB. On analysis of behavioral variables, HIV infection status of regular sex partner (*P* = 0.047, OR = 1.361, 95% confidence interval [*CI*]: 1.004–1.844), history of smoking (*P* = 0.023, OR = 2.273, 95% *CI*: 1.118–6.419) were significantly associated with active TB. On analysis of clinical variables, BMI (*P* = 0.044, OR = 0.553, 95% *CI*: 0.311–0.984), latest CD 4^+^ T-cell count (*P* = 0.002, *OR* = 3.714, 95% *CI*: 1.612–8.577), status of ART use (*P* = 0.000, *OR* = 3.889, 95% *CI*: 1.869–8.090) and duration of HIV infection (*P* = 0.005, *OR* = 2.282, 95% *CI*: 1.287–4.048) were significantly associated with active TB (Table 3).

**Table 3:** Characteristics of cases and controls.

Variable	Cases(n = 82) n(%)	Controls (n = 82) n(%)	*P* value	*OR*	*95% CI* (for *OR*)
Occupation					
Farmer	49 (59.8)	57 (69.5)	0.241	1.421	0.790–2.556
Non-farmer	33 (48.2)	25 (30.5)			
Ethnicity					
Han	45 (54.9)	48 (58.5)	0.725	1.383	0.226–8.451
Zhuang	35 (42.7)	31 (37.8)	0.572	1.699	0.271–10.661
Other*	2 (2.4)	3 (3.7)	-	-	-
Education status					
Illiterate	10 (12.2)	10 (12.2)	0.673	1.373	0.314–5.998
Primary school	38 (46.3)	33 (40.2)	0.387	1.566	0.566–4.350
Junior middle school	27 (32.9)	29 (35.4)	0.672	1.261	0.431–3.693
Above high school*	7 (8.5)	10 (12.2)	-	-	-
Marital status					
Married	22 (26.8)	30 (36.6)	0.136	1.800	0.831–3.899
Unmarried	60(73.2)	52 (63.4)			
Place of Residence					
Urban area	16 (19.5)	10 (12.2)	0.207	0.571	0.240–1.362
Rural area	66 (80.5)	72 (87.8)			
Local residence duration					
Less than 3 months	28 (34.1)	22 (26.8)	0.186	0.848	0.664–1.083
3–6 months	4 (4.9)	2 (2.4)			
6–12 months	1 (1.2)	1 (1.2)			
More than 12 months	49 (59.8)	57 (69.5)			
Household incomes per capita, Yuan					
Less than 1 000	12 (14.6)	12 (14.6)	1.000	1.000	0.489–2.046
1 000–10 000	66 (80.5)	66 (80.5)			
More than 10 000	4 (4.9)	4 (4.9)			
Number of family members					
none	6 (7.3)	12 (14.6)	0.512	0.897	0.648–1.241
two	21 (25.6)	14 (17.1)			
three	33 (40.2)	21 (25.6)			
Four or more	22 (26.8)	35 (42.7)			
History of close contact with TB patient					
No	78 (95.1)	80 (97.6)	0.423	2.000	0.366–10.919
Yes	4 (4.9)	2 (2.4)			
History of BCG vaccination					
No	58 (70.7)	55 (67.1)	0.602	0.833	0.420–1.653
Yes	24 (29.3)	27 (32.9)			
History of former plasma donors					
No	81 (98.8)	82 (100.0)	0.610	65.289	0.000–628084630.4
Yes	1 (1.2)	0 (0.0)			
History of intravenous drug					
No	74 (90.2)	74 (90.2)	1.000	1.000	0.375–2.664
Yes	8 (9.8)	8 (9.8)			
HIV infect status of regular sex partner					
Positive	11 (13.4)	20 (24.4)	0.027	0.306	0.107–0.876
Negative	17 (20.7)	17 (20.7)	0.331	0.646	0.268–1.560
Unclear	36 (43.9)	26 (31.7)	0.208	0.520	0.188–1.440
Null information*	18 (22.0)	19 (23.2)	-	-	-
Extramarital sex					
No	19 (23.2)	15 (18.3)	0.416	0.714	0.317–1.608
Yes	63 (76.8)	67 (81.7)			
Status of condom using					
Frequent	5 (6.1)	3 (3.7)	0.385	2.009	0.416–9.696
Sometimes	18 (22.0)	20 (24.4)	0.801	1.147	0.396–3.317
Never	40 (48.8)	36 (43.9)	0.413	1.42	0.610–3.338
Null information*	19 (23.2)	23 (28.0)	-	-	-
History of drinking					
No	68 (82.9)	66 (80.5)	0.695	0.857	0.396–1.853
Yes	14 (17.1)	16 (19.5)			
History of smoking					
No	52 (63.4)	66 (80.5)	0.023	2.273	1.118–4.619
Yes	30 (36.6)	16 (19.5)			
BMI					
Less than 18.5 kg/m^2^	36 (43.9)	27 (32.9)	0.044	0.553	0.311–0.984
18.5–24 kg/m^2^	44 (53.7)	47 (57.3)			
More than 24 kg/m^2^	2 (2.4)	8 (9.8)			
Chronic disease					
No	68 (82.9)	72 (87.8)	0.396	1.444	0.617–3.379
Yes	14 (17.1)	10 (12.2)			
Latest CD4^+^ T-cell count					
Less than 200 cell/mm^3^	27 (32.9)	46 (56.1)	0.002	3.714	1.612–8.577
More than 200 cell/mm^3^	55 (67.1)	36 (43.9)			
HIV Risk Factors					
Sexual	73 (89.0)	71 (86.6)	1.000	1.000	0.250–3.998
Intravenous drug	4 (4.9)	6 (7.3)	0.672	0.667	0.102–4.354
Other*	5 (6.1)	5 (6.1)	-	-	-
Status of ART					
No	25 (30.5)	51 (62.2)	0.000	3.889	1.869–8.090
Yes	57 (69.5)	31 (37.8)			
Duration of HIV infection					
Less than 5 years	13 (15.9)	21 (25.6)	0.005	2.282	1.287–4.048
5–9 years	45 (54.9)	52 (63.4)			
More than 9 years	24 (29.3)	9 (11.0)			

* reference for dummy raviable analysis

On multivariate analysis for significant variables from univariate analysis, latest CD 4^+^ T-cell count (*P* = 0.025, *OR* = 3.288, 95% *CI*: 1.161–9.311), use of ART (*P* = 0.000, *OR* = 7.775, 95% *CI*: 2.618–23.094), history of smoking (*P* = 0.052, *OR* = 2.996, 95% *CI* 0.992–9.053) and duration of HIV infection (*P* = 0.000, *OR* = 5.946, 95% *CI*: 2.221–15.915) were independent risk factors for active TB in people living with HIV/AIDS (Table 4).

**Table 4:** Adjusted analysis for factors associated with TB.

Variable	*B*	*Wald* value	*P* value	*OR*	*95% CI* (for *OR*)
History of smoking	1.097	3.784	0.052	2.996	0.992–9.053
Latest CD 4^+^ T-cell count	1.190	5.024	0.025	3.288	1.161–9.311
Status of ART	2.051	13.634	0.000	7.775	2.618–23.094
Duration of HIV infection	1.783	12.592	0.000	5.946	2.221–15.915

## Discussion

The risks factors associated with TB among people living with HIV/AIDS could generally be divided into two categories: biological and non-biological factors. It is clear about the biological factors. When MTB infects individuals infected with HIV, it can stimulate viral replication and accelerate HIV disease progression[10–12]. HIV infection can also make a person get active TB easier by inducing cytokines-Ⅱ[13]. However, external factors are very complicated. Social activities and environment can influence disease transmission and change their expected course.

WHO published HIV/AIDS prevention and antiretroviral treatment guidelines in 2015 and strongly recommended that all individuals with HIV/AIDS should receive ART as soon as possible, regardless of CD4^+^ cell count[14]. ART not only can reduce the incidence of opportunistic infectious diseases (including TB), but also can prevent the secondary transmission of HIV. After diagnosis of TB, individuals should initiate ART in two to 8 weeks. Some cohort studies have shown that TB incidence decreased by 70~90% in HIV-infected subjects receiving ART[15–16]. Unfortunately, the surveillance population in our study sites had lower rate of ART use (47.4%). It is probably because of the lack of health knowledge and poor conditions in rural area. The multivariate analysis also showed an obvious difference between the ART recipients and the ones who were not receiving ART. The risk of TB disease among those who had not received ART was about 8 times higher than those who had received it (*OR* = 7.775). This indicates that the most effective way to prevent active TB in people living with HIV/AIDS is initiating ART as soon as possible. Physicians should monitor the indicators closely during the process of treatment for the occurrence of immune reconstitution inflammatory syndrome related TB[17].

Our study shows the significant correlation between lower CD 4^+^ T-cell count and active TB among HIV infected patients, as has been previously reported[18]. The risk of TB disease among those with CD 4^+^ T-cell counts less than 200 was 3 times higher than those with higher levels of CD 4^+^ T-cells (*OR* = 3.288). impaired immunity creates the prerequisite for mycobacterium tuberculosis infection., proliferation and spread. We should note that the onset of TB in an individual infected with HIV is insidious because of the impaired immune response[19]. Physicians often misdiagnose TB in those with mild clinical signs and atypical medical imaging. Another result of our research identified a long duration of HIV infection as another independent risk factors (*OR* = 5.946). As an incurable disease, AIDS can destroy the body’s defense system step by step. But some opportunistic infections can be prevented and controlled, including TB. So we suggest that medical institutions to provide regular TB screening every year, and increase the frequency of clinical evaluation for patients with longer duration of HIV infection and in those living with low CD 4^+^ T-cell counts, regardless of the presence of clinical symptoms. It is also necessary for patients with LTBI to received isoniazid preventive therapy (IPT) to avoid active TB[20].

In this study, we also focus on behavioral and clinical factors for TB in HIV-patients. The result of multivariable analysis shows a significant correlation between smoking and active TB among HIV infected individuals. This the same result has been observed in West Africa[21], Thailand[22], India[23] and China[24]. The possibility of active TB among smokers was 3 times that of non-smoking subjects in our study (*OR* = 2.996). Although the correlation between smoking and active TB remains controversial, two meta-analysis provide more evidence for the association between smoking and active TB[25–26]. At present, China is the largest consumer of tobacco worldwide. There are 350million smoking adults, and about 1 million people die each year from smoking related diseases[27]. Burning of tobacco produces at least 5 different known human carcinogens and a variety of toxic substances that can damage the respiratory mucociliary transport system and block the body’s removal of mycobacterium tuberculosis. Therefore, medical workers should strengthen the health education aspect of their daily work. Great efforts should be made to inform patients the dreadful consequences of smoking and encourage smokers to quit.

This research detected active TB by multiple methods, including symptom screening, digital chest image, CT, sputum culture, Xpert-MTB/RIF Assay, IGRAs, histopathological examination and drug sensitivity or biochemical testing. This can help to increase the sensitivity for identifying active TB and LTBI in HIV-infected patients and excluding NTM. Thus we feel this result of prevalence rate is a credible estimate.

One meta-analysis of active TB prevalence in HIV-infected patients in Mainland China (including 29 studies) from 2010 showed that the average prevalence rate was 7.2%[28]. In our research this rate was 15.6%, two-fold higher than the reported average rate. And this rate also higher than that in Yunnan, Sichuan and Henan[29]. The HIV infection rate among new active TB patients was also higher than the average rate in China[30]. As the area has double the disease burden, the TB/HIV co-infection situation is severe. Although the TB prevalence rate in the general population of Guangxi (about 90 cases/100 000 py)[31–32] is higher than that in USA (3.4 case/100 000 in 2011) [33]. this rate among HIV/AIDS population was 173 times higher than that in the general population of Guangxi. For decades, few studies have investigated the risk factors for active TB among HIV-infected patients in Guangxi. It is necessary for us to find the causes for this situation.

## Limitations

There were some limitations in our research. First, as a pair-matched case-control study, it was difficult to select controls, especially in inpatient group. The inpatients with HIV were serious and maybe got another pulmonary opportunistic infectious disease except TB. So the individual with lung disease should be out. Next step, we may search the controls from outpatients. Second, as a retrospective study, there was a potential for bias and inaccurate data collection. A prospective cohort study would provide more complete data. Third, as a multivariable analysis, there may have been some risk factors we did not account for, such as awareness of TB and HIV, living environment and biological indicators.

## Conclusion

The prevalence of TB among people living with HIV/AIDS in Guangxi was 173 times higher than general population in Guangxi. This is a severe public health problem in Guangxi. It is necessary for the government to integrate the treatment and control of the two diseases for optimal planning and resource allocation. Further strategies for the prevention and treatment among TB/HIV co-infected patients should be implemented. According to the multivariate analysis, the risk factors for active TB among people living with HIV/AIDS were smoking, lower CD 4^+^ T-cell count, long duration of HIV-infection and the lack of ART. We suggest that medical and public health workers should strengthen health education of TB/HIV prevention and treatment, and support smoking cessation. High frequency of TB surveillance and early initiation of ART is necessary, as is IPT and infection control in healthcare facilities.
